# Evaluation of Reversed Administration Order of Busulfan (BU) and Cyclophosphamide (CY) as Conditioning on Liver Toxicity in Allogenic Hematopoietic Stem Cell Transplantation (ALL-HSCT)

**DOI:** 10.18502/ijhoscr.v14i3.3725

**Published:** 2020-07-01

**Authors:** Mani Ramzi, Nasrin Namdari, Shirin Haghighat, Hourvash Haghighinejad

**Affiliations:** 1 Hematology and Bone Marrow Transplant Research Center, Shiraz University of Medical Sciences, Shiraz, Iran; 2 Department of Hematology and Medical Oncology, Shiraz University of Medical Sciences, Shiraz, Iran; 3 Department of Hematology, Hematology Research Center, Medical Oncology and Stem Cell Transplantation, Shiraz University of Medical Sciences, Shiraz, Iran; 4 Department of Family Medicine, Shiraz University of Medical Sciences, Shiraz, Iran

**Keywords:** Allogeneic transplant, Conditioning, Busulfan, Cyclophosphamide

## Abstract

**Background:** Busulfan (BU) in combination with cyclophosphamide (CY) is used as an effective conditioning regimen in hematopoietic SCT. Busulfan, depletes glutathione level in liver and causes elevated levels of CY metabolites. Cyclophosphamide metabolites are highly toxic for sinusoidal endothelial cells and cause VOD/ SOS with high mortality rate.

**Materials and Methods:** Between September 2013 and September 2015, all adult patients with acute leukemia who were candidates for myeloablative allogenic SCT and were admitted to Stem Cell Transplantation center were enrolled in this prospective randomized clinical trial. We tested the hypothesis that reverse administration from BU-CY (n=28) to CY-BU group (n=27) would reduce liver toxicity.

**Results:** Liver function tests were significantly higher in the BU-CY group between day -1 and +4 (p<0.05), but VOD/SOS was not diagnosed in both groups. The incidence and severity of acute GVHD was higher in the BU-CY group, but not statistically significant. Engraftment and mortality rate were not different.

**Conclusion:** These data support the concept that CY-BU is associated with less liver toxicity, suggesting CY-BU is superior to BU-CY as conditioning.

## Introduction

 Hematopoietic Stem cell Transplantation is a curative therapy for a number of malignant and non-malignant disorders. Busulfan followed by Cyclophosphamide (BU-CY) is used as an effective conditioning regimen in allogeneic hematopoietic cell transplantation^1^. Liver toxicity and hepatic sinusoidal obstruction syndrome (SOS), also known as Hepatic Veno-occclusive disease (VOD), is a potentially life-threatening complication that can occur after myeloablative hematopoietic stem cell transplantation^2^^-^^5^. Incidence of VOD/SOS ranges from 8-14%, and its severe form is associated with mortality rate higher than 80%^6^.

VOD/SOS begins with injury to sinusoidal endothelial cells and hepatocytes due to toxic metabolites generated by high-dose alkylating chemotherapy conditioning regimens such as busulfan, cyclophosphamide, melphalan, 6-mercaptopurine and possibly thiotepa.^7^Busulfan and Cyclophosphamide are metabolized in liver^8^^,^^9^. Busulfan is not toxic for hepatocytes and sinusoidal endothelial cells, but metabolites of cyclophosphamide are highly toxic to sinusoidal endothelial cells^10^^-^^12^.

Cytotoxic effect of cyclophosphamide is mediated by its active metabolites such as 4-hydroxy cyclophosphamide (4-OHCP) and phosphoramide mustard (PM).The extent of cyclophosphamide metabolism depends in part on the activity and concentration of aldehyde dehydrogenase, glutathione and glutathione S-Transferase^13^^-^^15^. Glutathione is involved in busulfan clearance and starting conditioning regimens with busulfan depletes glutathione level in liver and causes elevated levels of cyclophosphamide metabolites^11^^-^^12^^,^^17^.

Restoration of hepatic and sinusoidal endothelial cell glutathione levels prevents injury to hepatic sinusoidalin in several different animal models of toxic liver injury^18^. Cyclophosphamide administration in less than 24 hours after the last dose of busulfan causes more mucositis and VOD/SOS because of negative effect on cyclophosphamide pharmacokinetics^19^. Studies have suggested less hepatotoxicity when busulfan is administered after cyclophosphamide^8^^,^^19^^-^^21^.

Here, we report a prospective randomized clinical trial designed to test the hypothesis that reverse administration of CY and BU reduces hepatotoxicity in comparison to standard BU-CY conditioning regimen.

## MATERIALS AND METHODS

 All adult patients with acute leukemia who were candidates for myeloablative allogeneic SCT and admitted to Stem Cell Transplantation center of Shiraz were enrolled in this prospective randomized clinical trial. They were referred to our hospital from September 2013 to September 2015. All eligible patients were provided written informed consent. All procedures were in accordance with the Helsinki protocol of 1975 and approved by Ethics Committee of Shiraz University of Medical Sciences (Shiraz, Iran).

**Inclusion criteria**

Eligibility criteria included: 1) patients with acute leukemia (ALL and AML) that needs Allogenic SCT 2) age ≥15 years 3) Karnofski performance status of >70% at the time of HCT

**Exclusion criteria**

1) HIV infection   2) chronic liver disease 3) active hepatitis

**Conditioning regimen**

Patients randomized to two groups: CY-BU and BU-CY. Patients in CY-BU group (n=27) received cyclophosphamide on days -8 through -6, and busulfan on days -5 through-2. Transplantation was done on day 0. Patients in the control group (BU-CY, n=28) received busulfan on days -8 through -5 and cyclophosphamide on days -4 through -2.

Cyclophosphamide was administered with a total dose of 120 mg/kg i.v and busulfan i.v with dose of 0.8 mg/kg/dose every 6 hours for a total 16 doses. Prophylactic phenytoin was given from days -8 through -2 to all patients. Graft-versus-Host-disease prophylaxis consisted of cyclosporine and metotheroxate. Cyclosporin was given from day -1 as an i.v route with 3mg/kg/d, with adjusted doses according to cyclosporine level. Cyclosporin was changed to oral route when the patients could tolerate oral dose. Metotheroxate was given at a dose of 10 mg/m^2^ i.v on days +1, +3, +6 and +11. All patients received antifungal, antiviral, antibacterial and ursodiol prophylaxis in accordance to standard practice.

We checked liver function tests on admission, before starting conditioning regimen, one day before transplantation, on transplantation day and on days +4, +8, +12, +16,+20 and +30. The patients were followed till days +100 for GVHD presentation and mortality. Neutrophil engraftment was defined first day of two consecutive days of PMN ≥500/dl.

**Statistical analyses**

Statistical comparison of engraftment between two groups was done by independent t-test. Frequency of GVHD was compared by Chi-square test between two groups. Comparison of the liver function tests between two groups was done using Mann-Whitney test.

## Results

 Patients and their characteristics are summarized in Table 1.The median age of patients in BU-CY group was 30 years (range, 15-61 years). Sixteen patients (57.1%) were male and 12(42.9%) were female. Thirteen patients (46.43%) had ALL, 13(46.43%) had AML and 2(7.14%) had MDS. The median age of patients in CY-BU group was 31 years (range: 20-53 years). Seventeen patients (63%) were male and 10 (37%) were female. Nine (33.3%) had ALL, 18(66.7%) had AML and none of them had MDS.

There was no statistically significant difference between the two groups in terms of age, sex and underlying disease distribution. All transplants were from HLA-matched sibling donors, except one who was HLA-matched from his child.

**Engraftment**

Median times to myeloid engraftment (BU-CY cohort 13 days (range: 9-17) vs CY-BU cohort 13 days (range: 10-18)) were similar between two groups (P: 0.93).

**Graft Versus Host Disease**

The cumulative incidence of acute GVHD at day +100 was 46.6% in the BU-CY group and 37% in the CY-BU group (p-value= 0.48). Severity of acute GVHD in the BU-CY group was 30.8% grade 1, 38.5% grade 2, 7.7% grade 3, and 23.1% grade 4. None of the patients in the CY-BU group had grade 1 and 4 GVHD, 90% had grade 2 and 10% grade 3.

Skin was the most frequent involved organ between the two groups. GVHD was more severe in the BU-CY group (38.4% grade 3 and 4) compared with the CY-BU group (10% grade 3 and 4).

**Liver toxicity**

**Figure 1:**
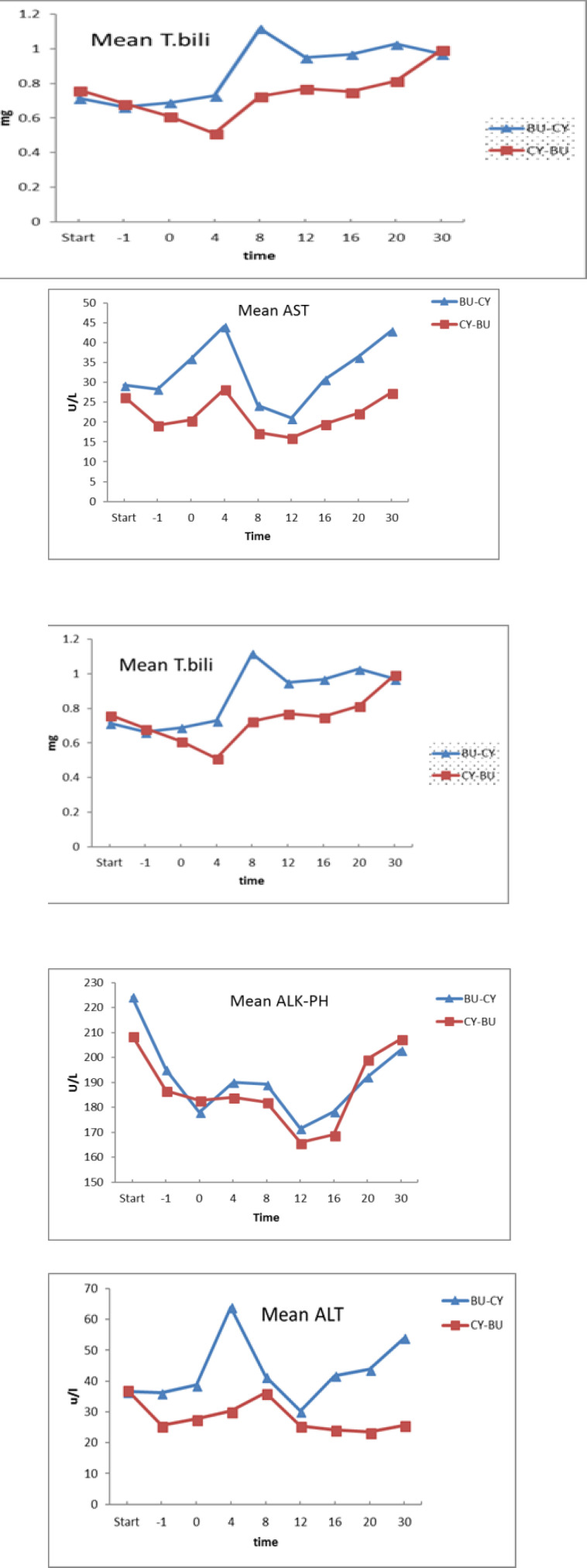
Mean serum levels of AST, ALT, T. bilirubin, D.bilirubin and Alkaline phosphatase for the BU-CY and CY-Bu group at day -8 (before starting the conditioning regimen), -1 (one day before transplantation), 0, +4, +8, +12, +16, +20 and +30.

Diagnosis of VOD /SOS was not made in both groups. Liver function tests including alkaline phosphatase and bilirubin showed no statistically significant difference between the two groups, but mean values of ALT on one day before (p=0.02) transplant day (p=0.025) and day +4 post-transplant (p=0.03) were significantly higher in the control group (Figure 1).

Mean values of AST on one day before (p=.001) and on transplant day (p=0.001) were significantly higher in the control group (Figure 1).

**Mortality**

The cumulative incidence of TRM at day +100 was 10.7% in BU-CY group and 3.7% in CY-BU group (p=0.32). Causes of TRM in the BU-CY group was relapse (n=1, 3.57%), GVHD (n=1, 3.57%) and GVHD simultaneous with CMV infection (n=1, 3.57%). The cause of TRM in the CY-BU group was GVHD (n=1, 3.7%).

**Table1:** Patients Characteristics

**Characteristics**	**BU-CY**	**CY-BU**	
Total Patients, n%	28	27	
Sex, n%			0.66
Male	16(57.1%)	17(63%)	
Female	12(42.9)	10(37%)	
Age			0.82
Median (range)	30(15-61)	31(20-53)	
Underlying disease, n%			0.8
All	12(44.4%)	9(33.3%)	
Aml	13(48.1%)	18(66.7%)	
MDs	2(7.4%)	0(0%)	
Donor, n%			0.36
Identical Sibling	26(96.3%)	27(100)	
Other family members	1(3.7%)	0(0%)	
Donor Sex, n%			0.25
Female in male	4(14.8%)	3(11.1%)	
Others	23(85.2%)	24(88.9%)	
Disease condition at Transplantation			0.11
First remission	15(55.6%)	20(74.1%)	
Second remission	3(11.1%)	3(11.1%)	
Others	9(14.8%)	4(14.8%)	

## Discussion

 One of the possible approaches to minimizing liver toxicity caused by BU-Cy conditioning is reversing the order of administration, giving CY followed by busulfan.

Median time to myeloid engraftment was not different between BU-CY and CY-BU group in our study (p= 0.93). Our results were comparable with those of Cantoni et al. and Kerbauy et al. Myeloid engraftment was similar in mice treated with BU-CY v/s CY-BU conditioning regimen^8^^,^^20^. It seems that reverse administration of conditioning regimen does not affect myeloid engraftment.

The incidence of acute GVHD was similar between the two groups (46.4% in BU-CY and 37% in CY-BU, p=0.48) in our study, but patients in the BU-CY group had more severe GVHD (grade 3 and 4) compared to the CY-BU group (38.4% vs 10%). Cantoni et al. demonstrated that patients in the BU-CY group had more acute GVHD compared to patients in the CY-BU group (75% vs 44%, p=0.001), but the severity of acute GVHD was similar between the two groups^8^.

Liver toxicity and VOD/SOS is a potentially life-threatening complication after myeloablative hematopoietic stem cell transplantation. In our study, mean values of AST and ALT were significantly higher in the BU-CY group in comparison to the CY-BU group (p<0.05). In the study by Cantoni et al., the incidence of SOS was significantly higher in the BU-CY group (12.5% vs 0% in CY-BU group) (p= 0.006). Moreover, serum levels of liver function tests were higher in patients with BU-CY conditioning regimen.^8^ In the study by Kerbauy et al., liver function tests were significantly higher in the BU-CY group (p<0.05) and similar results were reported^20^.

Rezvani et al. evaluated administering cyclophosphamide followed by intravenous targeted busulfan in patients with AML, MDS and myelofibrosis. Compared with BU-CY conditioned patients, CY-BU conditioned patients had greater exposure to cyclophosphamide and less exposure to hydroxycylophosphamide^21^.

In patients with myelofibrosis, the incidence of SOS was significantly lower in patients given CY-BU in comparison to BU-CY (0% vs 30%), whereas the incidence of SOS was low in both cohorts with AML and MDS. Rezvani et al. recommends CY-BU as a superior conditioning regimen in patients with myelofibrosis^21^.

## CONCLUSION

 According to our results and other studies, it seems that CY-BU conditioning is associated with less liver toxicity, VOD/SOS and acute GVHD without interfering with engraftment and mortality rate. Our finding which is in good concordance with previous studies showed that CY-BU is effective and less toxic; therefore, transplant centers can use it as a replacement choice of conditioning regimen.
